# Gallbladder Agenesis without Additional Biliary Tracts Abnormality

**DOI:** 10.1155/2022/3209658

**Published:** 2022-06-23

**Authors:** Seyed Mostafa Meshkati Yazd, Hamidreza Bayati, Sara Sadat Nabavizadeh, Reza Shahriarirad

**Affiliations:** ^1^Department of Surgery, Tehran University of Medical Sciences, Tehran, Iran; ^2^Department of Surgery, Iran University of Medical Sciences, Tehran, Iran; ^3^Student Research Committee, Shiraz University of Medical Sciences, Shiraz, Iran; ^4^Thoracic and Vascular Surgery Research Center, Shiraz University of Medical Science, Shiraz, Iran

## Abstract

**Background:**

Gallbladder agenesis (GA) is a very uncommon disorder of the biliary system. Diagnosis of GA can be difficult and may result in unnecessary procedures. In this case report, we will discuss our experience with an intraoperative accidental diagnosis of GA in a middle-aged woman that was effectively treated. *Case Presentation.* A 46-year-old woman presented with abdominal pain, nausea, vomiting, and intolerance to meals. Laparoscopic surgery was conducted based on sonographic imaging and a preliminary diagnosis of chronic cholecystitis. No gallbladder was seen during laparoscopy, and the patient was diagnosed as a case of GA. The laparoscopy was terminated, and the patient was referred for magnetic resonance cholangiopancreatography (MRCP) to confirm the diagnosis. Finally, endoscopic retrograde cholangiopancreatography (ERCP) and sphincterotomy were performed to alleviate symptoms. After one year of follow-up, the patient's overall condition is satisfactory and symptom-free.

**Conclusion:**

Our case exemplifies this common blunder. Therefore, we are reporting a case of GA discovered intraoperatively to increase surgeons' awareness and preparedness for this possible differential diagnosis and minimize unnecessary operational intervention.

## 1. Introduction

Gallbladder agenesis (GA) is a rare biliary system anatomical abnormality with an incidence rate of 0.01–0.075%. It is characterized by the absence of the gall bladder or cystic duct [1]. The actual incidence rate is unclear because most afflicted people present with no symptoms, and many are found inadvertently during unrelated procedures or autopsy [2]. Nonetheless, 50% of individuals may develop gallbladder pathology symptoms such as pain in the right upper quadrant, nausea or vomiting, intolerance to fatty foods, dyspepsia, and jaundice [3]. As a result, it becomes challenging to distinguish GA from other biliary illnesses. The disease's rarity and difficulty in preoperative diagnosis is a challenging issue that may result in unnecessary surgical intervention, a high risk of iatrogenic damage, and increased morbidity [4].

The following is a case report of a patient who presented with symptoms of biliary system involvement and was confirmed as GA during laparoscopy.

## 2. Case Presentation

We present the case of a recently treated 46-year-old woman with no significant medical history who came to the emergency department with approximately ten months of right upper quadrant pain described as sharp, intermittent, radiating to the back, and associated with nausea and vomiting. The pain was exacerbated by fatty meals without any change in bowel habits or urine symptoms. She denied having any additional medical conditions or a family history of gastrointestinal problems. On physical examination, she exhibited normal vital signs, minor abdominal pain in the upper abdomen, and no significantly abnormal routine laboratory data. Her baseline metabolic profile was normal, including an unremarkable liver function test.

**Figure 1:**
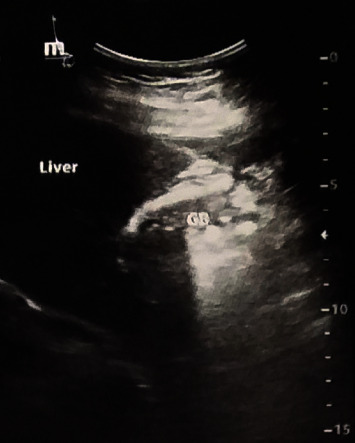
Scleroatrophic (shrunken) gallbladder with a normal biliary tree at abdominal ultrasonography.

The patient underwent an abdominal ultrasound in the right upper quadrant, revealing features consistent with chronic cholecystitis and a scleroatrophic (shrinking) gallbladder, or in other words, contracted and shrunken in size, which made it not visible during the ultrasonographic imaging test. The biliary tree was normal. At the same time, there was no sonographic evidence of Murphy's sign, gallbladder wall thickening (2 mm), pericholecystic fluid, or intra or extrahepatic biliary duct dilatation (Figure 1).

According to the clinical and radiological findings, laparoscopic cholecystectomy was performed with the preliminary diagnosis of chronic cholecystitis.

**Figure 2:**
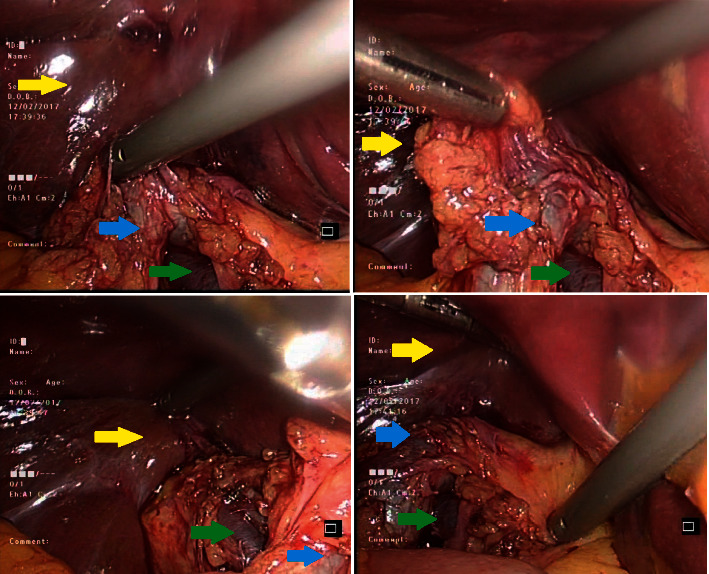
Intraoperative figures demonstrating liver (yellow arrow), common bile duct (blue arrow), and portal vein (green arrow).

Intraoperatively, the gallbladder could not be visualized from the junction of the left and right hepatic ducts until it disappeared behind the second part of the duodenum. There was no evidence of a gall bladder, cystic duct, or cystic artery (Figure 2 and supplementary file 1).

**Figure 3:**
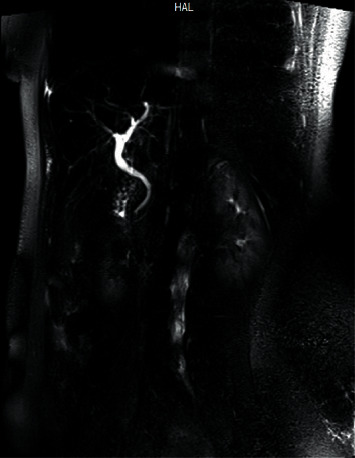
Magnetic resonance cholangiopancreatography during first-day postoperation, demonstrating normal intrahepatic bile ducts and slight dilation of common bile duct with an absence of gallbladder.

We concluded the operation at the laparoscopic level to avoid any unintended damage. Subsequently, on the first postoperative day, we performed magnetic resonance cholangiopancreatography (MRCP), which demonstrated normal liver structure, normal intrahepatic bile ducts, and slight dilatation of the common bile duct (CBD), with the absence of gallbladder (Figure 3). The patient underwent endoscopic retrograde cholangiopancreatography (ERCP) and sphincterotomy one day later to improve the remaining symptoms, based on the possibility of biliary tract dyskinesia. After treatment, the patient's symptoms alleviated over the next month, and the patient is still symptom-free one year later.

## 3. Discussion

GA, known as congenital absence of the gall bladder, is a rare developmental defect that affects less than one in every 6500 live births [5]. To our knowledge, Lemery et al. described the first case of GA in 1701. Since then, a few similar clinical cases have been recorded in the English medical literature. Along with its rarity, the disease's comparable symptoms and indistinguishable ultrasound imaging from other biliary illnesses contribute to its delayed identification [1, 3, 6].

The diagnosis is significantly delayed in most cases, resulting in adverse consequences from the unnecessary operation [7–9]. Our case exemplifies this common blunder. Therefore, we are reporting a case of GA discovered intraoperatively to increase surgeons' awareness and preparedness for this possible differential diagnosis and minimize unnecessary operational intervention. Differential diagnoses such as bile duct stenosis, Oddi sphincter stenosis, the presence of gallstones or sludge in the bile ducts, or bile duct dyskinesia are considered in these conditions.

Abdominal ultrasonography of the right upper quadrant is the standard initial assessment for patients presenting with biliary system involvement symptoms. Despite ultrasonography's high sensitivity for diagnosing biliary system diseases, GA cannot be reliably distinguished from a shrunken, constricted gallbladder, which may be accompanied by hyperechogenic shadows that were mistaken for chronic cholecystitis or cholelithiasis [10, 11]. Based on our patient's presence of biliary-related symptoms, we performed preoperative abdominal sonography, which revealed a scleroatrophic gallbladder with a normal biliary tree, increasing the suspicion of chronic cholecystitis. To avoid this common mistake, according to Malde's 2010 diagnostic and management algorithm for GA, in the absence of the WES triad (visualization of the gallbladder wall, stone echo, and acoustic shadow) and the presence of a contracted sclerotic gallbladder, additional imaging should be obtained before operative intervention to improve the accuracy of the diagnosis [11].

In the case of an intraoperative diagnosis of GA, the surgeon must rule out an ectopic gallbladder by exploring the ectopic gallbladder's most common sites. This procedure requires extensive surgical investigation, which increases the risk of biliary damage and the need for open surgery conversion. Most authors agree that if the gallbladder is not visible during laparoscopy, it is more beneficial to discontinue further surgical exploration and confirm the diagnosis with a comprehensive postoperative radiologic examination [12, 13].

MRCP is often considered the preferred diagnostic method. This noninvasive imaging technique allows a comprehensive evaluation of the biliary system. MRCP is the optimum imaging approach, since it accurately identifies GA before and after surgery and when ultrasonography is inconclusive and ruling out the possibility of an ectopic gallbladder. Additionally, computed tomography (CT) scanning and ERCP can be performed to determine GA. However, nonvisualization of the gallbladder is frequently attributed to a blocked cystic duct, anatomic differences, or technical errors, with GA being the least likely cause; therefore, they are preferred to be used as postoperative modalities if GA is suspected at laparoscopy [11, 14, 15]. After determining the possibility of GA during the laparoscopic procedure in our patient, we aborted the procedure and performed an MRCP to confirm the diagnosis. The results of MRCP dispelled our doubts about the biliary system's anatomical structure. It is worth mentioning that with the improvement of modern diagnostic imaging such as ultrasonography, CT, or MRI, diagnosing or at least suspecting GA has become less challenging. However, our experience regarding this case is that in cases with symptoms of biliary colic and an ultrasound report of chronic cholecystitis including contraction or shrunken gallbladder during preoperative evaluations, while ultrasound evidence was questionable, we recommend further evaluation with noninvasive imaging instruments associated with the gallbladder and bile ducts, including MRCP. Also, considering the possibility of GA in centers with limited imaging equipment and tertiary centers necessitates further evaluation and halting surgical interventions until a more precise diagnosis is achieved.

Due to the rarity of GA, there are no defined diagnosis and management guidelines. In individuals with mild to moderate symptoms, most experts recommend conservative therapy employing smooth muscle relaxants. Additionally, sphincterotomy should be performed in severe cases [3, 16]. In this case, she underwent ERCP and subsequent sphincterotomy due to the severity of the symptoms and failure to respond to muscle relaxants. Since patients with GA without biliary tract abnormality usually do not have biliary colic symptoms, the possibility of biliary tract dyskinesia should be considered.

## 4. Conclusion

GA is a rare congenital biliary defect that commonly resembles the clinical and radiologic features of more common biliary illnesses, demanding unnecessary surgical procedures. This situation may be averted in most cases by increasing surgeons' knowledge of this condition and proper use of radiologic techniques. In the event of a not visualized gallbladder in the ultrasound, a conservative strategy with follow-up imaging and preoperative MRCP is recommended. Furthermore, additional procedures should be avoided in cases detected during laparoscopy to prevent the morbidity associated with conversion to open surgery.

## Data Availability

The data used to support this study are included within the article and are available from the corresponding author upon request. A supplementary video file of the manuscript has been uploaded online at “https://drive.Google.com/file/d/1O8dgWTsK6ZRCc-9PBUPAXfyWYpiURl6I/view?usp=sharing.”

## Supplementary Materials

Supplementary Materials Supplementary Video 1. Intraoperative feed of patient with gallbladder agenesis without additional biliary tracts abnormality.
